# Electrocardiographic Findings and In-Hospital Mortality of COVID-19 Patients; a Retrospective Cohort Study

**DOI:** 10.22037/aaem.v9i1.1250

**Published:** 2021-06-12

**Authors:** Mohammad Haji Aghajani, Amirmohammad Toloui, Moazzameh Aghamohammadi, Asma Pourhoseingholi, Niloufar Taherpour, Mohammad Sistanizad, Arian Madani Neishaboori, Ziba Asadpoordezaki, Reza Miri

**Affiliations:** 1 Prevention of Cardiovascular Disease Research Center, Shahid Beheshti University of Medical Sciences, Tehran, Iran.; 2 Department of Cardiology, School of Medicine, Shahid Beheshti University of Medical Sciences, Tehran, Iran.; 3 Physiology Research Center, Iran University of Medical Sciences, Tehran, Iran.; 4 Department of Clinical Pharmacy, School of Pharmacy, Shahid Beheshti University of Medical Sciences, Tehran, Iran.; 5 Department of Psychology,Maynooth University, Kildare, Ireland.; 6 Kathleen Lonsdale Institute for Human Health Research,Maynooth University, Kildare, Ireland.; 7 Imam-Hussein Medical and Educational Centre, Shahid Beheshti University of Medical Sciences, Tehran, Iran

**Keywords:** Electrocardiography, Prognosis, Hospital mortality, COVID-19

## Abstract

**Background:**

Although current evidence points to the possible prognostic value of electrocardiographic (ECG) findings for in-hospital mortality of COVID-19 patients, most of these studies have been performed on a small sample size. In this study, our aim was to investigate the ECG changes as prognostic indicators of in-hospital mortality.

**Methods:**

In a retrospective cohort study, the findings of the first and the second ECGs of COVID-19 patients were extracted and changes in the ECGs were examined. Any abnormal finding in the second ECG that wasn’t present in the initial ECG at the time of admission was defined as an ECG change. ECGs were interpreted by a cardiologist and the prognostic value of abnormal ECG findings for in-hospital mortality of COVID-19 patients was evaluated using multivariate analysis and the report of the relative risk (RR).

**Results:**

Data of the ECGs recorded at the time of admission were extracted from the files of 893 patients; likewise, the second ECGs could be extracted from the records of 328 patients who had an initial ECG. The presence of sinus tachycardia (RR = 2.342; p <0.001), supraventricular arrhythmia (RR = 1.688; p = 0.001), ventricular arrhythmia (RR = 1.854; p = 0.011), interventricular conduction delays (RR = 1.608; p = 0.009), and abnormal R wave progression (RR = 1.766; p = 0.001) at the time of admission were independent prognostic factors for in-hospital mortality. In the second ECG, sinus tachycardia (RR = 2.222; p <0.001), supraventricular arrhythmia (RR = 1.632; p <0.001), abnormal R wave progression (RR = 2.151; p = 0.009), and abnormal T wave (RR = 1.590; p = 0.001) were also independent prognostic factors of in-hospital mortality. Moreover, by comparing the first and the second ECGs, it was found that the incidence of supraventricular arrhythmia (RR = 1.973; p = 0.005) and ST segment elevation/depression (RR = 2.296; p <0.001) during hospitalization (ECG novel changes) are two independent prognostic factors of in-hospital mortality in COVID-19 patients.

**Conclusion:**

Due to the fact that using electrocardiographic data is easy and accessible and it is easy to continuously monitor patients with this tool, ECGs can be useful in identifying high-risk COVID-19 patients for mortality.

## 1. Introduction

COVID-19 is the name of a disease, caused by the novel "Severe Acute Respiratory Syndrome Coronavirus 2", which appeared in December 2019 in Wuhan, China. Since the announcement of the global pandemic of COVID-19 until December 13, 2020, the number of infected people has exceeded 70 million cases and the death toll has exceeded 1.5 million people worldwide, according to the World Health Organization (1). Overall, COVID-19 can cause a range of symptoms in different patients, from a mild to a severe and fatal disease (2).

There are Studies demonstrating that COVID-19 is a multifactorial disease, affecting not only the lungs, but also the central nervous system, the cardiovascular system, and even the blood circulation system (3-6). The available clinical evidence suggests that current treatments are mostly symptomatic, and no definitive cure is yet available. The efficacy of current antiviral and nonsteroidal anti-inflammatory drugs is still questionable (7-9).

Since there is no definitive cure available for COVID-19, it may be possible to manage and monitor high-risk patients more accurately, and commence critical care by observing the red flags in patients from the beginning of disease. Several factors have been proposed for predicting the outcome of COVID-19 patients. Current findings indicate that older age and the presence of comorbidities such as hypertension, diabetes, and cardiovascular diseases are associated with COVID-19 severity, and the highest mortality rates have been observed in these groups of patients (10). Heart failure and cardiac arrest are among the most common causes of death in COVID-19 patients (11). Arrhythmias and electrocardiographic changes, both due to the administered drugs and as direct effects of the virus, have also been reported (12, 13).

In general, ECG is a very useful tool in diagnosing a variety of cardiac disorders. In most cases, electrocardiograms help in diagnosis of myocarditis, arrhythmias and heart failure (14). Due to changes in heart’s electrical activity in most cardiovascular diseases and its diagnostic value in cardiac damage, and since heart’s damage in the course of COVID-19 is associated with a high mortality rate, the assessment of ECG changes could be used in determining disease prognosis and management of patients (15). Although several studies have been performed to evaluate the prognostic value of electrocardiographic findings for mortality of COVID-19 patients, most of these studies have a small sample size and they only assess the relationship between electrocardiographic findings at the time of admission and patients’ overall mortality (12). Nonetheless, the effect of electrocardiographic changes during hospitalization on patients’ in-hospital mortality is not clear. Given the facts above, our aim in this study was to assess the value of changes in patients' ECGs as prognostic indicators of in-hospital mortality based on a study with large sample size.

## 2. Methods

***2.1. Study design and setting***

The present retrospective cohort study was performed on the records of patients who were admitted to Imam-Hossein Hospital in Tehran, between 18 February and 10 July 2020. The present study was approved by the ethics committee of Shahid Beheshti University of Medical Sciences (Ethics code: IR.SBMU.RETECH.REC.1399.681) and the researchers adhered to the principles of the Helsinki Convention.

***2.2. Subjects***

All patients with COVID-19 who had at least one ECG during their hospital stay were included in this study. COVID-19 infection was confirmed by a positive RT-PCR (polymerase chain reaction) test for severe acute respiratory syndrome coronavirus 2 (SARS-CoV-2) from a nasopharyngeal specimen (nasopharynx). Exclusion criteria were patients without sufficient information in their hospital records, patients without a recorded discharge status (dead or alive) or patients with known ECG abnormalities. 

***2.3. Data collection***

Baseline and demographic variables of patients were extracted from the hospital’s patient registration system. A total of 893 patients had at least one ECG during hospitalization and more evaluation of these records revealed that 328 patients had also a second ECG. ECGs were interpreted by a cardiologist, and to ensure data accuracy, ECGs were randomly re-examined by a senior cardiology attending. The findings of the first and the second ECGs were reviewed separately and recorded in the statistical program.

If an abnormal finding was repeated in at least two leads, it was included in the study as a definite abnormal finding. All ECGs were recorded by a 12 standard-lead electrocardiography tool. Electrocardiographic findings were sinus tachycardia, sinus bradycardia, supraventricular arrhythmia, ventricular arrhythmia, right bundle branch block (RBBB), left bundle branch block (LBBB), incomplete RBBB, incomplete LBBB, left anterior hemi-block, posterior Interventricular conduction delay (IVCD), bifascicular block, abnormal R Wave progression in precordial leads, presence of Q wave, prolonged QT interval, ST segment abnormalities, and abnormal T wave.

To evaluate ECG changes, any abnormal finding in the second ECG that wasn’t present in the initial one at the time of admission was defined as a change in the ECG; accordingly, abnormal findings were defined as any changes in the waves’ shape or differences in length or timing of the normal components of an ECG.

***2.4. Statistical analysis***

Continuous variables were described as mean ± standard deviation (SD), and categorical variables were expressed as counts (percentage). We have examined normality assumption by checking kurtosis, skewness, box plot and Q-Q plot. T-test and Mann–Whitney U test were used for comparisons of means of variables in alive and dead patients. Besides, for evaluating the association between categorical variables, Chi-square test and fisher’s exact test were used. In addition, a multivariate logistic regression model was performed for investigating the association of electrocardiographic findings and in-hospital mortality of COVID-19 patients. To avoid over-fitting in the multivariate model, only factors which had a p-value less than 0.1 in univariate analysis were selected for the multivariate model. Final model was selected according to backward Wald logistic regression. The findings were reported as odds ratio (OR) and 95% confidence interval (95% CI). Two-side P-value less than 0.05 was considered statistically significant. All analyses were done using Statistical Package for the Social Sciences (SPSS) 24.0.

## 3. Results

***3.1. Baseline characteristics***

The data of 893 ECGs at the time of admission was documented in patients' records and could be extracted; Of these, 494 patients were male (55.3%). The mean age of patients was 61.8±17.2 years (range: 10-99 years). The duration of hospitalization varied between 1 and 80 days (mean ± SD: 7.9 ± 6.8 days). 107 patients (12%) were admitted to the ICU and 231 patients (25.9%) finally passed away (Table 1).

***3.2. First ECG and in-hospital mortality***

According to the patients' records, 893 patients had an interpretable ECG at the time of admission. Examination of these ECGs showed that the most common abnormal findings in the ECG of COVID-19 patients at the time of admission were Sinus tachycardia (35.5%), abnormal T wave (24.7%), ST segment depression (19.1%), and prolonged QT interval (18.2%), bi-fascicular block (17.2%), and left anterior hemi-block (13.2%). Univariate analyses showed that age (p <0.001), sinus tachycardia (p <0.001), sinus bradycardia (p = 0.022), supraventricular (p <0.001) and ventricular (p = 0.037) arrhythmias, IVCD (p = 0.007), abnormal R wave progression in peri-cordial leads (p = 0.002), ST segment elevation / depression (p = 0.002), and abnormal T wave (p = 0.023) had a significant correlation with in-hospital mortality of COVID-19 patients (Table 1).

Multivariate analysis showed that increasing age (RR = 1.036, 95% CI: 1.029, 1.044; p <0.001) is one of the prognostic factors of in-hospital mortality in COVID-19 patients. Additionally, sinus tachycardia (RR = 2.342; 95% CI: 1.250, 2.280; p <0.001), supraventricular arrhythmia (RR = 1.688; 95% CI: 1.250, 2.280; p = 0.001), ventricular arrhythmia (RR = 1.854; 95 % CI: 1.154, 2.979; p = 0.011), IVCD (RR = 1.608; 95% CI: 1.129, 2.291; p = 0.009), and abnormal R Wave progression (RR = 1.766; 95% CI: 1.260, 2.474; p = 0.001) in the initial ECG at the time of admission were independent prognostic factors of in-hospital mortality (Table 2).

***3.3. Second ECG and in-hospital mortality***

Examination of patients’ records showed that 328 patients underwent a second ECG examination during their hospital stay. The most common abnormal findings on the second ECGs were abnormal T wave (31.1%), sinus tachycardia (30.5%), ST segment depression (22.6%), prolonged QT interval (20.1%), bifascicular block (16.1). %), supraventricular arrhythmia (11.9%), left anterior hemi-block (11.3%), and sinus bradycardia (10.7%). Univariate analyses illustrated that the presence of sinus tachycardia (p = 0.001), sinus bradycardia (p = 0.011), supraventricular arrhythmia (p <0.001), ST segment elevation / depression (p = 0.037), and abnormal T wave (p = 0.003) in the second ECG of patients had a significant correlation with their in-hospital mortality. Moreover, the correlation between mortality of patients and the presence of IVCD (p = 0.065) and abnormal R wave progression in peri-cordial leads (p = 0.059) in the second ECG was also close to the significance level (Table 3).

Multivariate analysis showed that older age (RR = 1.022, 95% CI: 1.014, 1.031; p <0.001) is still one of the independent prognostic factors of in-hospital mortality in COVID-19 patients. Likewise, the presence of sinus tachycardia (RR = 2.222; 95% CI: 1.597, 3.091; p <0.001), supraventricular arrhythmia (RR = 1.632; 95% CI: 1.792, 3.866; p <0.001), abnormal R Wave progression (RR = 2.151) 95% CI: 1.206, 3.834; p = 0.009), and abnormal T wave (RR = 1.590; 95% CI: 1.221, 2.069; p = 0.001) in the second ECG were independent prognostic factors of in-hospital mortality (Table 4).

***3.4. Electrocardiographic changes during hospitalization and in-hospital mortality***

Data of 328 patients were analysed in this section. By comparing the second ECG with the ECG at the time of admission, it was found that the most common changes in electrocardiograms during hospitalization were sinus tachycardia (11.5%), prolonged QT interval (9.0%), sinus bradycardia (6.7%), ST segment elevation/depression (4.3%), abnormal T wave (4.0%), supraventricular arrhythmia (4.0%), and ventricular arrhythmia (3.4%), respectively. Univariate analyses showed that the incidence of supraventricular arrhythmia (p = 0.029) and ST segment elevation/depression (p=0.006) during hospitalization has a strong correlation with patients’ in-hospital mortality (Table 5).

Moreover, multivariate analysis showed that supraventricular arrhythmia (RR = 1.973; 95% CI: 1.234, 3.154; p = 0.005) and ST segment elevation/depression (RR = 2.296; 95% CI: 1.574, 3.349; p < 0.001) during hospitalization, were two independent prognostic factors of in-hospital mortality in COVID-19 patients (Table 6).

## 4. Discussion

This retrospective cohort is one of the few studies with a large sample size, which investigates the prognostic value of COVID-19 patients’ ECG findings in predicting their in-hospital mortality. The findings of the present study disclosed that abnormal changes in the ECG, both at the time of admission and during hospital stay can be used for predicting disease prognosis. The analyses were performed in three sections. In the first part, the relationship between electrocardiographic findings at the time of admission and in-hospital mortality of COVID-19 patients was studied. In the second part, the relationship between in-hospital mortality and abnormal findings in the second ECG of patients during hospitalization was investigated. Finally, the relationship between the in-hospital mortality of COVID-19 patients and the changes that occurred between the first and the second ECG was investigated. An interesting point obtained from all three sections of the analysis is the proof of the prognostic role of supraventricular arrhythmia in predicting in-hospital mortality of COVID-19 patients. It was also found that the presence of sinus tachycardia and abnormal R Wave progression in precordial leads, both in the first and the second ECG of patients has a significant independent relationship with in-hospital mortality in COVID-19 patients. Finally, the presence of abnormal T wave in the second ECG or ST segment elevation/depression during hospitalization is a prognostic factor for mortality of COVID-19 patients.

In the present study, supraventricular arrhythmia consisted of atrial fibrillation, atrial flutter, premature atrial contraction, atrial tachycardia, and multifocal atrial tachycardia. In all three parts of analysis, it was found that supraventricular arrhythmia has a significant and independent relationship with mortality in patients with COVID-19. Numerous studies have shown that the occurrence of supraventricular arrhythmias, especially atrial fibrillation, increases the risk of stroke, heart attack, heart failure, and sudden cardiac death by increasing the risk of thrombosis. To be further illustrated, sudden cardiac death is the most common cause of cardiac death in patients with atrial fibrillation (16). In a situation with increased pressure on the cardiovascular system, due to hyperactivity of the immune system or infection, the occurrence of atrial arrhythmias with a risk of thrombosis, increases the risk of fatal cardiovascular events; and It must be taken into consideration that COVID-19 itself, especially in its severe forms, also rigorously increases the risk of thrombosis (17). Moreover, the risk of complications from atrial fibrillation such as stroke and thrombosis increase in the setting of other underlying diseases such as dyslipidaemia and diabetes, which have also been shown to be associated with more severe COVID-19 (18). There are other studies that show the association of other types of supraventricular arrhythmias, such as premature atrial contraction, with patient mortality (19).

The occurrence of abnormal R Wave progression in the precordial leads can point to 4 different causes: anterior myocardial infarction, left ventricular hypertrophy, right ventricular hypertrophy, and a natural variant in people whose anterior cardiac forces are weaker than others. Abnormal R Wave progression is expected to be more frequently detected in severe COVID-19; since in most cases, more severe COVID-19 usually occurs in the presence of other comorbidities such as diabetes and coronary heart disease, and these underlying diseases themselves could cause abnormal R Wave progression (20, 21). Considering the fact that abnormal R Wave progression is an independent prognostic factor in predicting patients’ in-hospital mortality, the emergence of this finding in patients' ECGs could warn physicians of the need for more accurate patient management.

Sinus tachycardia is common in patients with severe medical conditions and is significantly associated with COVID-19 patients’ mortality. A patient with severe COVID-19 may develop sinus tachycardia due to fever, systemic inflammation, shortness of breath, hypoxia, and dehydration. The presence of untreated sinus tachycardia can lead to ischemia of the heart, decreased cardiac output, cardiomyopathy, cardiac arrest, and death (22, 23). Therefore, sinus tachycardia seems to be a warning sign that the patient's condition could be getting worse and the patient is developing a more severe form of COVID-19; accordingly, sinus tachycardia can be used as an indicator in management of COVID-19 patients.

Abnormal T wave was another finding that was directly related to in-hospital mortality of COVID-19 patients. T wave inversion has been reported in 27% of patients with myocarditis and this T-wave was associated with cardiac edema in the corresponding position on cardiac MRI (24). Nonetheless, in delayed contrast enhancement imaging, performed to examine cardiac fibrosis, no correlation with T wave inversion was observed, which suggests its emergence in the acute phase of myocarditis and cardiac edema (25). Therefore, it seems that the presence of abnormal T wave in patients with COVID-19 may be due to acute myocardial injury as a result of the virus directly attacking the heart tissue, which could seriously affect the outcome of disease. More comprehensive studies are needed to prove this hypothesis.

The analyses of the present study showed that the occurrence of ST segment elevation/depression during hospital stay in a patient who had a normal ST segment at the time of admission, could be an alarm sign of their poor prognosis. The occurrence of ST segment elevation/depression during hospitalization can be due to virus’ direct attack to myocardial tissue, side effects of therapeutic agents used for patients, or an indicator of myocardial ischemia (26).

This study, like other retrospective studies, had its limitations. First, due to the recent pandemic, access to patients’ previous ECGs taken before the onset of COVID-19 was not possible and they were not included in this study. Second, other diagnostic tools such as echocardiography and laboratory tests were not used along with the ECG, as a result, the prognostic value of ECG may be over or underestimated. Moreover, due to the short follow-up period of patients (only during hospitalization), some of the changes that occurred after the second ECG were not included in this study.

**Table 1:** Baseline characteristics and first electrocardiographic (ECG) findings of COVID-19 patients

**Variable**	**Alive** **(n=662)**	**Dead** **(n=231)**	**Total** **(n=893)**	**P**
**Age (year; mean ± SD)**	58.4±16.9	71.4±13.9	61.8±17.2	<0.001
**Sex**				
Women	307 (46.4)	92 (39.8)	399 (44.7)	0.85
Men	355 (53.6)	139 (60.2)	494 (55.3)	
**ECG findings**				
**Sinus tachycardia**				
No	449 (67.8)	127 (55.0)	576 (64.5)	<0.001
Yes	213 (32.2)	104 (45.0)	317 (35.5)	
**Sinus bradycardia**				
No	614 (92.7)	224 (97.0)	838 (93.8)	0.022
Yes	48 (7.3)	7 (3.0)	55 (6.2)	
Supraventricular arrhythmia*				
No	616 (93.1)	189 (81.8)	805 (90.1)	<0.001
Yes	46 (6.9)	42 (18.2)	88 (9.9)	
**Ventricular arrhythmia** ^#^				
No	646 (97.6)	219 (94.8)	865 (96.9)	0.037
Yes	16 (2.4)	12 (5.2)	28 (3.1)	
**RBBB**				
No	633 (95.6)	219 (94.8)	852 (95.4)	0.611
Yes	29 (4.4)	12 (5.2)	41 (4.6)	
**LBBB**				
No	644 (97.3)	223 (96.5)	867 (97.1)	0.562
Yes	18 (2.7)	8 (3.5)	26 (2.9)	
**Incomplete RBBB**				
No	643 (97.1)	225 (97.4)	868 (97.2)	0.829
Yes	19 (2.9)	6 (2.6)	25 (2.8)	
**Incomplete LBBB**				
No	656 (99.1)	226 (97.8)	882 (98.8)	0.306
Yes	6 (0.9)	5 (2.2)	11 (1.2)	
**Left anterior hemi-block**				
No	570 (86.1)	205 (88.7)	775 (86.8)	0.307
Yes	92 (13.9)	26 (11.3)	118 (13.2)	
**Left posterior hemi-block**				
No	662 (100.0)	229 (99.1)	891 (99.8)	0.067
Yes	0 (0.0)	2 (0.9)	2 (0.2)	
**IVCD**				
No	648 (97.9)	218 (94.4)	866 (97.0)	0.007
Yes	14 (2.1)	13 (5.6)	27 (3.0)	
**Bifascicular block**				
No	546 (82.5)	193 (83.5)	739 (82.8)	0.710
Yes	116 (17.5)	38 (16.5)	154 (17.2)	
**Abnormal R wave progression**				
No	639 (96.5)	211 (91.3)	850 (95.2)	0.002
Yes	23 (3.5)	20 (8.7)	43 (4.8)	
**Q wave in inferior leads**				
No	624 (94.3)	219 (94.8)	843 (94.4)	0.756
Yes	38 (5.7)	12 (5.2)	50 (5.6)	
**Q wave in lateral leads**				
No	659 (99.5)	231 (100.0)	890 (99.7)	0.573
Yes	3 (0.5)	0 (0.0)	3 (0.3)	
**Q wave in precordial leads**				
No	642 (97.0)	224 (97.0)	866 (97.0)	0.994
Yes	20 (3.0)	7 (3.0)	27 (3.0)	
**Prolonged QT interval**				
No	548 (82.9)	181 (78.7)	729 (81.8)	0.154
Yes	113 (17.1)	49 (21.3)	162 (18.2)	
**ST segment**				
Normal	527 (79.6)	159 (68.8)	686 (76.8)	0.002
Elevation	21 (3.2)	15 (6.5)	36 (4.0)	
Depression	114 (17.2)	57 (24.7)	171 (19.1)	
**Abnormal T wave**				
No	511 (77.2)	161 (69.7)	672 (75.3)	0.023
Yes	151 (22.8)	31.7 (30.3)	221 (24.7)	

IVCD: Interventricular conduction delay; LBBB: Left bundle branch block; RBBB: Right bundle branch block; SD: Standard deviation.

*, Supraventricular arrhythmia includes atrial fibrillation, atrial flutter, premature atrial contraction, atrial tachycardia, and multifocal atrial tachycardia.

#, Ventricular arrhythmia includes premature ventricular contraction, and ventricular tachycardia.

**Table 2:** Multivariate analysis of abnormal finding in first electrocardiography and in-hospital mortality of COVID-19 patients

**Variable**	**Relative risk**	**95% CI**	**P**
Male sex	1.110	0.913-1.350	0.292
Increase in age	1.036	1.029-1.044	<0.001
Sinus tachycardia	2.342	1.84-2.982	<0.001
Supraventricular arrhythmia*	1.688	1.250-2.280	0.001
Ventricular arrhythmia^#^	1.854	1.154-2.979	0.011
IVCD	1.608	1.129-2.291	0.009
Abnormal R wave progression	1.766	1.260-2.474	0.001
Abnormal T wave	1.108	0.909-1.350	0.308

CI: Confidence interval; IVCD: Interventricular conduction delay.

*, Supraventricular arrhythmia includes atrial fibrillation, atrial flutter, premature atrial contraction, atrial tachycardia, and multifocal atrial tachycardia.

#, Ventricular arrhythmia includes premature ventricular contraction and ventricular tachycardia.

**Table 3:** Second electrocardiographic findings of COVID-19 patients

**Variable**	**Alive** **(n=218)**	**Dead** **(n=110)**	**Total** **(n=328)**	**P**
**Sinus tachycardia**				
No	165 (75.7)	63 (57.3)	228 (69.5)	0.001
Yes	53 (24.3)	47 (42.7)	100 (30.5)	
**Sinus bradycardia**				
No	188 (86.2)	105 (95.5)	293 (89.3)	0.011
Yes	30 (13.8)	5 (4.5)	35 (10.7)	
Supraventricular arrhythmia*				
No	203 (93.1)	86 (78.2)	289 (88.1)	<0.001
Yes	15 (6.9)	24 (21.8)	39 (11.9)	
**Ventricular arrhythmia** ^#^				
No	213 (97.7)	104 (94.5)	317 (96.6)	0.191
Yes	5 (2.3)	6 (5.5)	11 (3.4)	
**RBBB**				
No	208 (95.4)	103 (93.6)	311 (94.8)	0.493
Yes	10 (4.6)	7 (6.4)	17 (5.2)	
**LBBB**				
No	209 (95.9)	102 (92.7)	311 (94.8)	0.225
Yes	9 (4.1)	8 (7.3)	17 (5.2)	
**Incomplete RBBB**				
No	215 (98.6)	109 (99.1)	324 (98.8)	0.716
Yes	3 (1.4)	1 (0.9)	4 (1.2)	
**Incomplete LBBB**				
No	214 (98.2)	108 (98.2)	322 (98.2)	>0.999
Yes	4 (1.8)	2 (1.8)	6 (1.8)	
**Left anterior hemi-block**				
No	194 (89.0)	97 (88.2)	291 (88.7)	0.827
Yes	24 (11.0)	13 (11.8)	37 (11.3)	
**Left posterior hemi-block**				
No	218 (100.0)	108 (98.2)	326 (99.4)	0.112
Yes	0 (0.0)	2 (1.8)	2 (0.6)	
**IVCD**				
No	215 (98.6)	104 (94.5)	319 (97.3)	0.065
Yes	3 (1.4)	6 (5.5)	9 (2.7)	
**Bifascicular block**				
No	186 (84.9)	90 (81.8)	276 (83.9)	0.469
Yes	33 (15.1)	20 (18.2)	53 (16.1)	
**Abnormal R wave progression**				
No	208 (95.4)	99 (90.0)	307 (93.6)	0.059
Yes	10 (4.6)	11 (10.0)	21 (6.4)	
**Q wave in precordial leads**				
No	206 (94.5)	106 (96.4)	312 (95.1)	0.458
Yes	12 (5.5)	4 (3.6)	16 (4.9)	
**Prolonged QT interval**				
No	171 (79.2)	87 (81.3)	258 (79.9)	0.651
Yes	45 (20.8)	20 (18.7)	65 (20.1)	
**ST segment**				
Normal	163 (74.8)	70 (63.6)	233 (71.0)	0.037
Elevation	15 (6.9)	6 (5.5)	21 (6.4)	
Depression	40 (18.3)	34 (30.9)	74 (22.6)	
**Abnormal T wave**				
No	162 (74.3)	64 (58.2)	226 (68.9)	0.003
Yes	56 (25.7)	46 (41.8)	102 (31.1)	

IVCD: Interventricular conduction delay; LBBB: Left bundle branch block; RBBB: Right bundle branch block; SD: Standard deviation.

*, Supraventricular arrhythmia includes atrial fibrillation, atrial flutter, premature atrial contraction, atrial tachycardia, and multifocal atrial tachycardia.

#, Ventricular arrhythmia includes premature ventricular contraction, and ventricular tachycardia.

**Table 4:** Multivariate analysis of abnormal finding in second electrocardiography and in-hospital mortality of COVID-19 patients

**Variable**	**Relative risk**	**95% CI**	**P**
Increase in age	1.022	1.014-1.031	<0.001
Sinus tachycardia	2.222	1.597-3.0915	<0.001
Supraventricular arrhythmia*	1.632	1.792-3.866	<0.001
Ventricular arrhythmia^#^	1.510	0.754 -3.022	0.244
Abnormal R wave progression	2.151	1.206-3.834	0.009
Abnormal T wave	1.590	1.221-2.069	0.001

CI: Confidence interval

*, Supraventricular arrhythmia includes atrial fibrillation, atrial flutter, premature atrial contraction, atrial tachycardia, and multifocal atrial tachycardia.

#, Ventricular arrhythmia includes premature ventricular contraction, and ventricular tachycardia.

**Table 5:** Changes in clectrocardiographic findings during hospitalization in COVID-19 patients

**Variable**	**Alive** **(n=218)**	**Dead** **(n=110)**	**Total** **(n=328)**	**P**
**Sinus tachycardia**				
No	193 (88.5)	99 (90.0)	292 (89.0)	0.688
Yes	25 (11.5)	11 (10.0)	36 (11.5)	
**Sinus bradycardia**				
No	200 (91.7)	106 (96.4)	306 (93.3)	0.114
Yes	18 (8.3)	4 (3.6)	22 (6.7)	
Supraventricular arrhythmia*				
No	213 (97.7)	102 (92.7)	315 (96.0)	0.029
Yes	5 (2.3)	8 (7.3)	13 (4.0)	
**Ventricular arrhythmia** ^#^				
No	213 (97.7)	106 (96.4)	319 (97.3)	0.483
Yes	5 (2.3)	4 (3.6)	11 (3.4)	
**LBBB**				
No	215 (98.6)	106 (96.4)	321 (97.9)	0.174
Yes	3 (1.4)	4 (3.6)	7 (2.1)	
**Left anterior hemi-block**				
No	215 (98.6)	106 (96.4)	321 (97.9)	0.174
Yes	3 (1.4)	4 (3.6)	7 (2.1)	
**Bifascicular block **				
No	212 (97.2)	105 (95.5)	317 (96.6)	0.394
Yes	6 (2.8)	5 (4.5)	11 (3.4)	
**Prolong QT interval**				
No	197 (91.2)	97 (90.7)	294 (91.0)	0.871
Yes	19 (8.8)	10 (9.3)	29 (9.0)	
**Abnormal R wave progression**				
No	214 (98.2)	106 (96.4)	320 (97.6)	0.449
Yes	4 (1.8)	4 (3.6)	8 (2.4)	
**ST segment**				
No	214 (98.2)	100 (90.9)	314 (95.7)	0.006
Yes	4 (1.8)	10 (91)	14 (4.3)	
**Abnormal T wave**				
No	212 (97.2)	103 (93.6)	315 (96.0)	0.137
Yes	6 (2.8)	7 (6.4)	13 (4.0)	

LBBB: Left bundle branch block.

*, Supraventricular arrhythmia includes atrial fibrillation, atrial flutter, premature atrial contraction, atrial tachycardia, and multifocal atrial tachycardia.

#, Ventricular arrhythmia includes premature ventricular contraction, and ventricular tachycardia

**Table 6:** Multivariate analysis of changes in electrocardiographic findings during hospitalization and in-hospital mortality of COVID-19 patients

**Variable**	**Relative risk**	**95% CI**	**P**
Supraventricular arrhythmia	1.973	1.234-3.154	0.005
ST elevation/depression	2.296	1.574-3.349	<0.001

CI: Confidence interval.

*, Supraventricular arrhythmia includes atrial fibrillation, atrial flutter, premature atrial contraction, atrial tachycardia, and multifocal atrial tachycardia.

## 5. Conclusion

The findings of the present study showed that abnormal changes in ECG, both at the time of admission and during hospitalization, can be very useful in predicting the prognosis of COVID-19. Supraventricular arrhythmia, sinus tachycardia, and abnormal R wave progression in precordial leads, in both of patients’ ECGs had a significant independent relationship with in-hospital mortality. Abnormal T wave in the second ECG or the presence of ST-segment elevation/depression during hospitalization can have a good prognostic role in predicting the mortality of COVID-19 patients. Therefore, considering the fact that measuring the electrical activity of heart is a cheap and accessible method and it is easy to continuously monitor patients with this tool, ECGs can be useful in identifying high-risk COVID-19 patients and giving them more medical care.

## 6. Declarations

### 6.1. Acknowledgement

The authors would like to thank the ICU and CCU medical and nursing personnel of Imam Hossein Hospital, Shahid Beheshti University of Medical Sciences; Dr. Ainaz Samadi, Dr. Amir Heydari, Dr. Fatemeh Nasiri, Dr. Mahboubeh Ghazanfarabadi, Faezeh Nesaei, Faezeh Fakour, Ghazaleh Aman-Abadi, Ghodsi Najjari, Golnoush Mortezaei, Maedeh Sayyad, and Vida Torabi who participated in this project and helped us in performing this project.

### 6.2. Authors' contribution

Study design: RM, MHA

Data gathering: MHA, MA, AP, NT, MS, ZA

Data analysis: RM, AP

Interpreting the findings: All authors

Manuscript writing: All authors

### 6.3. Funding and supports

This research has been supported and funded by Shahid Beheshti university of medical sciences.

### 6.4. Conflict of interest

The authors declare no conflict of interests.
